# Psychiatric services utilization in completed suicides of a youth centres population

**DOI:** 10.1186/1471-244X-6-36

**Published:** 2006-08-23

**Authors:** Johanne Renaud, François Chagnon, Bogdan Balan, Gustavo Turecki, Alexander McGirr, Claude Marquette

**Affiliations:** 1 Department of Child and Adolescent Psychiatry, CHU mère-enfant Sainte-Justine, 3175 Côte Sainte-Catherine, Université de Montréal, Montreal, Canada; 2 Université du Québec à Montréal, Montreal, Canada; 3 McGill Group for Suicide Studies, Douglas Hospital Research Center, McGill University, Montreal, Canada

## Abstract

**Background:**

From a retrospective study of youth centres (YCs) and coroner's files, we investigated youths' history of medical service utilization who died by suicide. This is the second of two papers on YCs population, the first paper having shown that the rate of psychopathology was higher in the YCs population compared to the general adolescent population.

**Methods:**

From 1995 to 2000, 422 youths, aged 18 years and younger, died as a result of suicide in Quebec. More than one-third received services from YCs at some point. Using the provincial physician payment and hospitalization database, we examined physical and psychiatric service utilization according to time intervals, as well as hospitalization for psychiatric reasons in the individuals' lifetime and in the year preceding suicide. Suicides were matched to living YCs youths for age, sex, and geographic area. YCs controls were then subdivided into two groups based on file information pertaining to the presence or absence of suicidal behavior or ideation.

**Results:**

Compared to living YCs youths, suicides had a higher rate of psychiatric service utilization in the week, month, 90 days, and year preceding suicide, as well as higher levels of lifetime hospitalization for psychiatric reasons than controls with or without a history of suicidal behavior or ideation. We found that 28.3% YCs suicides made use of psychiatric services in the year preceding suicide.

**Conclusion:**

The rate of psychiatric service utilization by YCs youth suicides is substantially inferior to the needs of this population. Our study underscores the need for appropriate recognition of psychiatric and suicidal problems among YCs population by social and psycho-educational professionals. At the same time, it highlights the issues of general practitioners' risk identification, psychiatric referral and treatment. Our findings suggest the need for improved organization and coordination of psychiatric services to ameliorate treatment delivery.

## Background

The rate of suicide of those receiving services under the responsibility of youth protection and rehabilitation services is reported to be 5.5 to 11 times higher than that of the adolescent population in general [1]. In the Province of Quebec, child welfare services, social services, rehabilitation programs and juvenile detention for troubled youths are provided by a single agency, comprised of sixteen regional branches. This agency has been dubbed Youth Centres (YCs).

This is the second of two companion papers on the YCs population. The first reported that the rate of psychopathology was higher in the YCs population compared to the general adolescent population [2]. Given higher rates of both 1) suicide and 2) psychopathology among YCs suicides, we were interested in studying these individuals' utilization of medical services. More specifically, we were interested in examining the rate of service utilization for psychiatric reasons and suicidality before suicide.

As such, this paper outlines the history of medical services utilization in a sample of Quebec adolescent suicides that were treated in YCs services.

## Methods

### Study sample

The sample has been described in detail in a previous communication [2]. Subjects were 12 through 18 years of age. Of the 143 subjects who were eligible for the study, 53 had an available YCs file, other files having been cleared according to the YC archival regulations. These subjects were randomly matched to three living subjects receiving YCs services at the time of the death of the index case, for age, sex and geographic area. This strategy allowed us to identify living YCs controls with and without file references to suicidal behavior or ideation, a dichotomy permitting higher degrees of specificity in analyses. We identified 135 subjects with available YCs files (107 boys and 28 girls), while 24 files were not available. From these 135 files, 40 revealed references to a history of suicidal behavior or ideation, while 95 revealed no such mention.

### Measures

For each suicide, the following information was extracted from the Quebec Coroner's Office computerized database: date of birth, sex, date of event (i.e. date of suicide), date of death, and means of suicide. For each subject (suicide and control groups), names and health insurance numbers were used for retrieving information about medical services from the physician fee-for-service database of the "Régie de l'assurance maladie du Québec" (RAMQ), a database regrouping information on medical interventions. Information pertaining to hospitalizations (reason for admission, length of stay, International Classification of Diseases Ninth Revision (ICD-9) diagnosis, and number of hospitalizations) was obtained from the Quebec Ministry of Health and Social Services (MHSS) hospitalization database system "Maintenance et Exploitation des Données pour l'Ètude de la Clientèle Hospitalière" (MED-ECHO). These data were retrieved for subjects' lifetime and for the one-year period preceding suicide or index period, using the discharge date in case of hospitalizations.

The utilization unit for outpatient medical services, "medical visit", regroups all outpatient services provided by a single physician to a single patient during one calendar day. A medical visit was classified as "psychiatric" if it included at least one mental health evaluation/intervention or otherwise, the visit was classified as "physical". The visits were further sub classified according to different time intervals: past week, past month, past 90 days, or past year. Hospitalizations from the MED-ECHO database were classified as "psychiatric" if they included at least one mental health diagnosis, or otherwise, as "physical". Analysis of the ICD-9 codes led to the exclusion of two incidences hospitalization related to fatal suicide attempts.

### Analysis

Data originating coming from the sources of information employed for this study have been found to be reliable [3,4]. All data were entered into a relational database, and analyses were carried out with the SPSS statistical package [5]. Each type of visit and hospitalization, according to time intervals, have been analyzed as present or absent for each subject by group (dichotomized variables) in order to perform non parametric analyses on numbers of subjects by category. We considered results as significant when the probability of type I error was equal or lower than 5%.

### Ethics

This study was approved by the local Institutional Review Board and by the Commission d'accès à l'information du Québec.

## Results

The 53 suicide cases who had available YC files comprised 42 boys and 11 girls (M: F ratio of 3.8:1), similar to the general YCs population male-female ratio for youth aged 10–19 years (3.6:1) [6]. The mean age was 16.8 years (girls 16.98 ± 1.4; boys 15.96 ± 1.9). The characteristics of the group are described in more detail in our previous paper [2]. As mentioned in our previous paper [2], 52.8% (n = 28) of the subjects were receiving YCs services at the time of their death, 20.0% had received YCs services in the past 6 months while 91.1% had received those services in the past 12 months.

### Physical Medical Visits

We found a higher proportion of "physical" medical visits among suicides in the week before death than among the two control groups (n = 11, 20.8% *vs*. n = 2, 5.0% *vs*. n = 8, 8.4%; X^2 ^= 7.15, df = 2, p < 0.05). In the month, 90 days, and year preceding index period or suicide, there were no significant differences across the three groups for physical medical visits (Table 1).

**Table 1:** Type of visit and hospitalization by time intervals and groups.

**Type of visit**		Subjects by Group*			
		
		**Suicide (N = 53)**	**Suicidal (N = 40)**	**Non suicidal (N = 95)**	**Chi2**
**Last week**	**Physical**	11	2	8	X^2 ^= 7.16, df = 2, p < 0.05
	**%**	20.8	5.0	8.4	
	**Psychiatric**	5.0	0.0	0.0	X^2 ^= 13.08, df = 2 p < 0.001
	**%**	9.4	0.0	0.0	
					
**Last 30 days**	**Physical**	16	10	21	NS
	**%**	30.2	25.0	22.1	
	**Psychiatric**	7	0	1	X^2 ^= 14.59, df = 2 p < 0.001
	**%**	13.2	0.0	1.1	
					
**Last 90 days**	**Physical**	25	18	38	NS
	**%**	47.2	45.0	40.0	
	**Psychiatric**	8	1	3	X^2 ^= 9.39, df = 2 p < 0.01
	**%**	15.1	2.5	3.2	
					
**Last year**	**Physical**	43	34	71	NS
	**%**	81.1	85.0	74.7	
	**Psychiatric**	15	6	6	X^2 ^= 13.38, df = 2 p < 0.001
	**%**	28.3	15.0	6.3	
					
**Past year Hospitalization**	**Total**	10	3	7	NS
	**%**	18.9	7.5	7.4	
	**Psychiatric**	9	2	1	X^2 ^= 14.6, df = 2 p = 0.001
	**%**	17.0	5.0	1.1	
**Lifetime Hospitalization**	**Total**	21	9	29	NS
	**%**	39.6	22.5	30.5	
	**Psychiatric**	15	5	4	X^2 ^= 17.7, df = 2 p < 0.001
	**%**	28.3	12.5	4.2	

* Number of Subjects by group that had at least one visit in the given time interval

### Psychiatric Medical Visits

In terms of psychiatric visits, we found a higher rate of service utilization for the suicide group in all four time intervals: last week (n = 5, 9.4% *vs*. n = 0, 0% *vs*. n = 0, 0%; X^2 ^= 13.08, df = 2, p < 0.001), last month (n = 7, 13.2% *vs*. n = 0, 0.0% *vs*. n = 1, 1.1%; X^2 ^= 14.59, df = 2, p < 0.001), last 90 days (n = 8, 15.1% *vs*. n = 1, 2.5% *vs*. n = 3, 3.2%; X^2 ^= 9.39, df = 2, p < 0.01), and last year (n = 15, 28.3% *vs*. n = 6, 15.0% *vs*. n = 6, 6.3%; X^2 ^= 13.38, df = 2, p < 0.001).

### Rates of hospitalization

We found a higher rate of lifetime (n = 15, 28.3% *vs*. n = 5, 12.5% *vs*. n = 4, 4.2%; X^2 ^= 17.7, df = 2, p < 0.001), and last year (n = 9, 17.0% *vs*. n = 2, 5.0% *vs*. n = 1, 1.1%; X^2 ^= 14.6, df = 2, p < 0.001) hospitalizations for psychiatric reasons in the suicide group compared to the control groups. In addition, 4 suicide subjects had been hospitalized after a suicide attempt between 7 months and 3 years prior to fatality. In terms of past year and lifetime hospitalizations (pooled physical and psychiatric), we found no significant difference across the groups.

### Gender differences

Exploratory analyses investigated gender differences in service utilization among cases and controls, respectively. In the suicide group, there were no significant differences between girls and boys for all indicators. Conversely, in the controls, there was a general trend in all indicators, girls having significantly more physical visits in the last week, last month and last 90 days (Table 2).

**Table 2:** Type of visit by group and sex.

			**Boys**	**Girls**	**Chi2**
**Suicides**	**Physical**	**Last 7 days**	8 (19.04%)	3 (27.27%)	NS
		**Last 30 days**	10 (23.8%)	6 (54.5%)	NS
		**Last 90 days**	19 (45.2%)	6 (54.5%)	NS
		**Last year**	34 (80%)	9 (81%)	NS
	**Psychiatric**	**Last 7 days**	4 (9,52%)	1 (9.09%)	NS
		**Last 30 days**	5 (11.9%)	2 (18.1%)	NS
		**Last 90 days**	6 (14.2%)	2 (18.1%)	NS
		**Last year**	11 (26.1%)	4 (36.3%)	NS
**Controls**	**Physical**	**Last 7 days**	4 (3.73%)	6 (21.42%)	X^2 ^= 10.12 p < 0.01
		**Last 30 days**	20 (18.6%)	11 (39.2%)	X^2 ^= 5.32 p < 0.05
		**Last 90 days**	40 (37.3%)	16 (57.1%)	X^2 ^= 3.57 p < 0.05
		**Last year**	83 (77.5%)	22 (78.5%)	NS
	**Psychiatric**	**Last 7 days**	0	0	NS
		**Last 30 days**	1 (0.9%)	0	NS
		**Last 90 days**	2 (1.86%)	2 (7.14%)	NS
		**Last year**	8 (7.47%)	4 (14.28%)	NS

## Discussion

In our study, we found higher rates of psychiatric medical visits and lifetime hospitalization for psychiatric reasons prior to suicide in our YCs sample who completed suicide. However, our results need to be interpreted with caution due to the following limitations: 1) groups were not matched for recency of YCs services; 2) our controls were not completely randomly selected, since they were first matched and then split into two groups on the basis of file references to suicidality; 3) some files were missing; 4) analyses were performed as group comparisons and not paired-comparisons between the case and the control; and 5) the suicidal or non-suicidal status of controls was based on file evidence, yet the absence of file evidence may not indicate absence of suicidality.

In our suicide group, 28.3% of youths showed psychiatric medical visits in the year preceding suicide, while between 9.0% and 15.0% had received psychiatric medical visits in the prior 3 months, month, and week. The rate of 28.3% in the YCs suicide group was higher compared to the 15.0% and the 8.9% found in the two control groups, and compared to the rate of 12.0% among unselected Quebec youth suicides, yet this rate remains insufficient [4]. Possible hypotheses for the insufficiency include more pernicious psychopathology and psychosocial-familial stressors among the YCs population. At the same time, interventions may have been inadequate or poorly integrated with community services, either during treatment or after discharge.

Nevertheless, studies have repeatedly found that medical and mental health services were present at some point, though not always at optimal levels, in youths who died by suicide [7-12]. Otherwise, the high rate of 81.1% of medical physical services in YCs suicide group as well as in controls, was quite similar to the rate of services in the suicide general population of youths of 78.0% [4].

These results raise two important issues: a better recognition of psychiatric and suicidal problems among YCs population, as well as referral to medical services by social and psycho-educational professionals. Furthermore, it highlights the problem of identification of youths at risk of suicide by general practitioners, youths to be referred to the psychiatrists or youths treated by the general practitioner de facto. It also underlines the challenge of improved organization (availability, type of services offered, etc.) of specialized psychiatric services and coordination among health services in order to ameliorate treatment delivery and to decrease the morbid risks in the YCs population.

In terms of clinical implications, identifying risk factors is very important, but in clinical contexts, there are too many false positives and false negatives for this to aid in the identification of high levels of risk for suicide. Current thinking suggests that while such risk factors should be borne in mind, ensuring high quality mental health care (including appropriate assessment of proximal and distal risks and appropriate follow-up) for all patients is likely to be the best approach to suicide prevention in a clinical setting [11].

## Conclusion

Future studies should be designed to focus on specific trajectories of service utilization in this population, including direct interviews with family members as well as comprehensive examinations of medical, psychosocial, school and psychiatric files. In conclusion, our study supports the International Association of Suicide Prevention' recommendation that improved treatment and management strategies for young people with suicidal problems and psychopathology may be very useful to prevent suicide [13,14], with increased accuracy in terms of risk factors and targeted-interventions.

## List of abbreviations

YC Youth Centre

RAMQ Régie de l'Assurance Maladie du Québec

MED-ECHO Maintenance et Exploitation des Données pour l'Étude de la Clientèle Hospitalière

ICD-9 International Classification of Diseases Ninth Revision

## Competing interests

This study was funded by the Direction de la planification stratégique et de l'évaluation du Ministère de la Santé et des Services sociaux du Québec, the Desjardins Sécurité Financière, and l'Association des centres jeunesse du Québec.

Dr Renaud was supported by a Canadian Institutes of Health Research Young Investigator award.

## Authors' contributions

JR and FC have made substantial contributions to conception and design, acquisition of data, analysis and interpretation of data, and involved in drafting the manuscript; BB has made substantial contributions to data analysis and interpretation of data; JR, FC, GT, AM, and CM have been involved in revising manuscript critically for important intellectual content, and all authors read and approved the final version to be published.

## Pre-publication history

The pre-publication history for this paper can be accessed here:


